# ‘Talk to me’: a mixed methods study on preferred physician behaviours during end‐of‐life communication from the patient perspective

**DOI:** 10.1111/hex.12384

**Published:** 2015-07-14

**Authors:** Amane Abdul‐Razzak, Diana Sherifali, John You, Jessica Simon, Kevin Brazil

**Affiliations:** ^1^ Department of OncologyUniversity of CalgaryCalgaryABCanada; ^2^ School of NursingMcMaster UniversityHamiltonONCanada; ^3^ Department of MedicineMcMaster UniversityHamiltonONCanada; ^4^ School of Nursing and MidwiferyQueen's University BelfastBelfastUK

**Keywords:** advance care planning, end‐of‐life communication, mixed methods, patient preference, physician–patient relations

## Abstract

**Background:**

Despite the recognized importance of end‐of‐life (EOL) communication between patients and physicians, the extent and quality of such communication is lacking.

**Objective:**

We sought to understand patient perspectives on physician behaviours during EOL communication.

**Design:**

In this mixed methods study, we conducted quantitative and qualitative strands and then merged data sets during a mixed methods analysis phase. In the quantitative strand, we used the quality of communication tool (QOC) to measure physician behaviours that predict global rating of satisfaction in EOL communication skills, while in the qualitative strand we conducted semi‐structured interviews. During the mixed methods analysis, we compared and contrasted qualitative and quantitative data.

**Setting and Participants:**

Seriously ill inpatients at three tertiary care hospitals in Canada.

**Results:**

We found convergence between qualitative and quantitative strands: patients desire candid information from their physician and a sense of familiarity. The quantitative results (*n* = 132) suggest a paucity of certain EOL communication behaviours in this seriously ill population with a limited prognosis. The qualitative findings (*n* = 16) suggest that at times, physicians did not engage in EOL communication despite patient readiness, while sometimes this may represent an appropriate deferral after assessment of a patient's lack of readiness.

**Conclusions:**

Avoidance of certain EOL topics may not always be a failure if it is a result of an assessment of lack of patient readiness. This has implications for future tool development: a measure could be built in to assess whether physician behaviours align with patient readiness.

## Introduction

With an ageing population, there is a pressing need to understand more about how to effectively communicate with people about their future health‐care wishes in a manner that preserves their dignity and autonomy and is satisfactory from the patient perspective. High‐quality end‐of‐life (EOL) communication has been associated with reduced costs and improved quality of care in the final days of life,^1^, ^2^ and terminally ill patients have identified a physician's ability to communicate about topics such as death and dying as a major priority to good EOL care.^3^

Previous studies suggest that two of the greatest opportunities to improve EOL care relate to patient–physician communication and patient engagement in EOL decision making.^4^, ^5^ There is currently no standard definition of EOL communication, but previous work has focused on the distinction between advance care planning (i.e. anticipatory planning for future personal and healthcare decisions in the context of one's values), vs. more immediate ‘in the moment’ decision making about treatment preferences in the context of a serious illness.^6^ In addition, a conceptual framework of EOL communication was recently developed by means of literature review and a survey of multidisciplinary Canadian experts using a modified Delphi method. This framework includes three domains: (i) advance care planning, including conversations about values and appointment of a substitute decision‐maker; (ii) goals of care decisions, including treatment preferences; and (iii) documentation, including personal directives and documentation of resuscitation preferences in medical charts.^7^ For the purposes of this study, EOL communication can be understood to broadly encompass the advance care planning and goals of care decisions activities described in these papers and may also include related information‐sharing processes.

Despite the recognition of its importance, studies indicate that the extent and quality of EOL communication is low. For example, a cross‐sectional survey conducted in the Netherlands showed that patients with advanced CHF or COPD were able to state their preferences on many EOL decisions, but that most had never discussed these items with their physician.^8^ Similarly, in a multicentre audit of EOL communication at 12 Canadian hospitals with 278 seriously ill patients and 225 family members, participants endorsed low levels of engagement in EOL communication with physicians and high levels of discordance (70%) between patients' stated preferences for EOL care and the preferences documented in the hospital chart (e.g. full cardiopulmonary resuscitation (CPR) vs. comfort care).^9^ The value of high‐quality EOL communication and the apparent paucity of such conversations creates an impetus to further study and understand how this situation can be improved.

The objective of this study was to understand patient perspectives on physician behaviours that help or hinder EOL communication. End‐of‐life communication is a complex topic that involves psychological, social and contextual factors that are well suited to examination using qualitative methods to understand why certain behaviours are important and in what contexts. However, given the widespread implications of good EOL communication for patients, healthcare providers and the healthcare system at large, data that are representative of a typical population with life‐limiting illness are also desirable. Given these two competing needs, we conducted a convergent parallel mixed methods study^10^ in which the qualitative and quantitative strands each contribute unique knowledge that, in combination, provide a more comprehensive understanding. The objective of the quantitative strand is to measure which behaviours appear to be most predictive of satisfaction with a physician's EOL communication skills, as rated by seriously ill inpatient participants. The objective of the qualitative strand was to more broadly understand seriously ill inpatients' perspectives on physician behaviours during EOL communication. Finally, during a separate mixed methods phase, we merged the qualitative and quantitative data for the purpose of elaboration and comparison – to examine how the data from each strand might complement or diverge from one another. This paper will focus on the quantitative and mixed methods analyses, as previously published work has described the qualitative strand in more detail.^11^

## Methods

**Figure 1:**
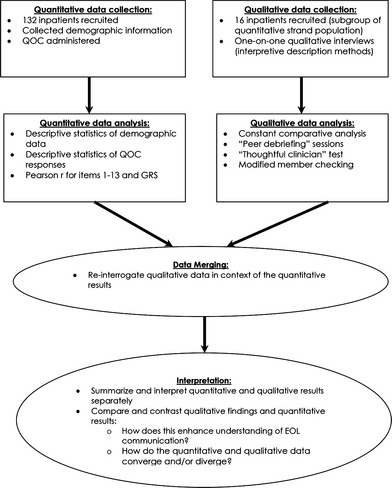
Mixed methods study flow diagram, which provides pictorial representation of the study design and conduct.

In this convergent parallel mixed methods design, we simultaneously conducted the qualitative and quantitative strands. The study design can be described as having independent implementation of research questions, data collection and analyses, with merging of the quantitative and qualitative results during a separate mixed methods analysis (see Fig. 1). The quantitative and qualitative strands were given equal priority in this study: data from both strands were considered to have equal weight and were merged for the purposes of comparison and elaboration.^10^, ^12^

### Recruitment

We recruited medical inpatients from three academic tertiary hospitals in Hamilton, Ontario and Calgary, Alberta from October 2012 to August 2013. We recruited patients to the quantitative strand (cross‐sectional survey) of the mixed methods study using inclusion criteria that identify a population of seriously ill medical inpatients aged 55 years or older with an estimated 6‐ to 12‐month mortality risk of 50% (see Table 1), similar to criteria used in previously published studies.^4^, ^5^ Patients were excluded from the quantitative strand if they were cognitively impaired (dementia or delirium as documented in health records, or healthcare team or research nurse assessment), unable to speak or read English, too fatigued or sick to participate, admitted for <48 h or could not recall any previous EOL communication encounters with a physician. For our semi‐structured qualitative interviews, we used a maximum variation sampling technique,^13^ aiming to recruit a population with different combinations of the following demographic variables: race (Caucasian vs. non‐Caucasian), gender and diagnosis (cancer vs. non‐cancer). This strategy is supported by the literature, which suggests that Caucasians and females are more likely to participate in EOL communication and that the uncertain prognoses associated with non‐cancer illnesses pose a greater barrier to EOL communication in comparison with cancer illness.^14^, ^15^, ^16^ Institutional research ethics board approval was obtained at each site prior to the initiation of study recruitment. Verbal and written informed consent was obtained from each participant.

**Table 1:** Selection criteria

*Inclusion criteria*
Age ≥55 and at least one of the following:
Hospital admission for congestive heart failure (CHF) with New York Heart Association (NYHA) Class IV symptoms or left ventricular ejection fraction (LVEF) ≤25%.
Hospital admission for severe chronic obstructive pulmonary disease (COPD) with one or more of the following: body mass index (BMI) <21; an exacerbation requiring hospitalization over the past year; shortness of breath causing the patient to stop walking after 100 m or after a few minutes on level ground; forced expiratory volume in 1 s (FEV_1_) ≤30% predicted; or partial pressure of carbon dioxide (PaCO_2_) ≥45 torr.
Hospital admission for liver cirrhosis with at least one of the following: history of hepatic coma; Child's class C liver disease or Child's class B liver disease with gastrointestinal bleeding.
Hospital admission for issue related to active metastatic cancer.
OR
ANY medical inpatient ≥80 years of age.
OR
Any medical inpatient for whom a physician answers ‘no’ to the following ‘surprise’ question: ‘Would you be surprised if this patient died within the next year?’*
*Exclusion criteria*
Unable to read and speak the English language.
Cognitive impairment, including mild cognitive impairment, dementia of any type or delirium. This was determined by review of the medical charts or clinical assessment by the research nurse or physician.
Patient has not had any discussions with a physician related to advance care planning and/or their wishes for care at the end of life
Hospitalization time of <48 h
Unable to participate for other reasons:
Participant fatigued or too sick
Healthcare team member feels that patient is not appropriate for enrolment

### Quantitative strand

We conducted a cross‐sectional study in which the quality of communication (QOC) questionnaire was administered to measure patients' self‐rated satisfaction with their physicians' EOL communication skills. The QOC is a patient‐reported 14‐item instrument that addresses aspects of a physician's EOL communication behaviours. This instrument was developed and has undergone validation work with a variety of samples of healthcare providers (i.e. physicians, nurses and social workers), and palliative care patients (i.e. advanced chronic obstructive pulmonary disease, AIDS and cancer) in the USA (both inpatients and outpatients). Factor analysis identified two subscales within the questionnaire: one that pertains to general communication skills (items 1–6) and one pertaining to EOL‐specific items (items 7–13).^3^, ^17^, ^18^, ^19^, ^20^, ^21^, ^22^ Each item asks the participant to rate his or her physician's skills in performing a specific behaviour, such as ‘when talking with Doctor “X” about important issues like becoming very ill, how good is he/she at using words that you can understand?’ The last item is a global rating score (GRS) that asks participants to rate their physicians' overall EOL communication skill level. For all items, the responses range from 0, which corresponds to ‘the very worst I could imagine’, to 10, ‘the very best I could imagine’, with an option to respond ‘don't know’ or ‘didn't do’. The QOC questions are posted in a web appendix. Items with a response of ‘don't know’ were treated as missing values while ‘didn't do’ responses were coded as zero. The latter coding strategy is suggested by tool authors,^23^ and we used this coding strategy because we speculated *a priori* that the lack of occurrence of certain items can be interpreted as suboptimal EOL communication.

We collected demographic data from patients' charts regarding age, gender, primary diagnosis (criterion by which a participant met study eligibility) and asked patients directly about number of hospitalizations over the past year, educational and ethnic background. Mean and standard deviation were reported for normally distributed continuous data, median and range were reported for non‐normally distributed continuous data, and frequency and proportion were reported for categorical data. In our primary analysis, we calculated the Pearson correlation coefficient (*r*) between individual QOC items and the global rating of skill (GRS) to determine which behaviours appear to be most predictive of the GRS. We used Fisher's transformation test to calculate whether there were statistically significant differences between the *r* values of items 1–6 (and the GRS). Given that 15 pairwise comparisons are made, the Bonferroni correction for multiple hypothesis testing yields a minimum *P* value of 0.003 to claim a statistically significant difference. IBM SPSS Statistics version 21 software was used for all statistical analyses.^24^

Sample size calculation was based on the primary analysis, and assumed a minimum Pearson correlation coefficient between each item and the GRS of 0.5, with a power of 0.9 and alpha of 0.05. A conservative correction for multiple hypotheses testing, by means of the Bonferroni method, was used to account for the fact that the correlation between each of 13 items and the GRS was being calculated.^25^ Based on these parameters, the required sample size was 118.

### Qualitative strand

The methods and findings for the qualitative strand have been previously reported elsewhere in more depth; here, we present a brief overview.^11^ We used interpretive description methods to explore seriously ill patients' perspectives of physician behaviours during EOL communication through 16 in‐depth, one‐on‐one interviews. Rather than focusing on the creation of theoretical frameworks, interpretive description is a qualitative method that is employed for the purpose of generating practical clinical knowledge that can be used by healthcare professionals.^26^ Although all participants had past EOL communication encounters with a physician, in some cases they described their hypothetical preferences when specific physician behaviours were not encountered.

One of the authors (AA) conducted all interviews to ensure consistency in approach. In the interview guide, we used questions designed to elicit physician behaviours that participants found helpful or harmful during EOL communication. We used a constant comparative approach, in which new data were compared to emerging themes from previous interviews to allow for further understanding of concepts and refinement of themes.^26^, ^27^, ^28^ To enhance the validity of results, all transcripts were read individually by each of two authors (AA and DS) and then consensus was reached on the categorization of data into themes, based on literal and interpretive meanings. Further, rigour was established in the qualitative strand through the use of techniques specific to interpretive description, including the ‘thoughtful clinician test’,^26^, ^29^ where evolving findings were reviewed with a physician with clinical experience in EOL communication (JY) to assess congruence with his past encounters. In addition, we employed ‘credibility checks’, a type of modified member‐checking strategy whereby evolving themes generated from previous interviews are discussed with participants to assess whether these align with their personal perspectives.^26^

### Mixed methods phase

During the mixed methods analysis, the qualitative data were interrogated once again by two authors (AA and DS) by reading through transcripts to identify data that could lend a complementary understanding of the quantitative results. In addition, we sought out data in which qualitative and qualitative findings appeared to be discrepant, as this can lead to new insights about the topic under study.^9^

## Results

### Quantitative results

**Figure 2:**
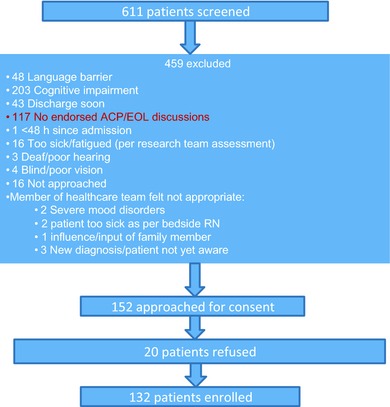
Recruitment flow diagram, which illustrates the recruitment strategy and results.

A total of 611 patients were identified as being eligible to participate in the quantitative strand of the study. Of this group, 348 patients were excluded for reasons relating to suitability (e.g. fatigue), an imminently planned discharge, and communication challenges (e.g. language barriers or cognitive impairment) (see Fig. 2). A total of 117 patients were excluded because they endorsed having no previous EOL‐related discussions with any physician, including no recollection of discussion about resuscitation preferences during the current hospitalization. Of the remaining 152 eligible patients who were approached, 132 consented, resulting in an enrolment rate of 86.8%.

As shown in Table 2, the mean age of participants was 80.3 years, and these participants experienced a mean of 2.4 hospitalizations over the past year (SD 1.4). More than half (64.4%) of the participants were female, the majority were Caucasian, and 47.7% of the participants were included in the study because they were medical inpatients aged 80 years or older. Almost one‐fourth (23.5%) of participants were eligible because of a diagnosis of active metastatic cancer.

**Table 2:** Quantitative strand participant demographics (*n* = 132)

Characteristic	No. of participants (%)
Mean age in years (SD): 80.3 (10.0)	
Mean no. hospitalizations/year (SD): 2.4 (1.4)	
Female	85 (64.4)
Education level	
Elementary school	57 (43.2)
High school diploma	42 (31.8)
Postsecondary degree or diploma	30 (22.7)
Missing	3 (2.3)
Ethnicity	
Caucasian	129 (97.7)
Non‐Caucasian	3 (2.3)
Reason for inclusion	
Congestive heart failure	16 (12)
Chronic obstructive pulmonary disease	18 (13.6)
Liver cirrhosis	1 (1)
Active metastatic cancer	31 (23.5)
Medical inpatient ≥80 years old	63 (47.7)
MD answered ‘no’ to surprise question	3 (2.3)

For the QOC items 1 to 6, which comprised the general communication subscale, the median scores ranged from 7 to 8 on a scale of 0 to 10 (see Table 3). For items 7–13, which comprised the EOL‐specific communication subscale, there were a large number of ‘didn't do’ responses (which, as discussed, were coded as zero), with median scores between 0 and 7 on a scale of 0–10.

**Table 3:** Descriptive Statistics for Quality of Communication tool responses

Item	*N*	‘didn't do’ responses (%)	Median and range	Pearson *r* for each item and GRI (*P* value)
1. Words you understand	131	0 (0)	8 (1–10)	0.56 (<0.001)
2. Looking you in the eye	122	1 (1)	8 (0–10)	0.52 (<0.001)
3. Answering all questions	132	8 (6)	8 (0–10)	0.59 (<0.001)
4. Listening	132	1 (1)	8 (0–10	0.53 (<0.001)
5. Caring	131	1 (1)	7 (0–10)	0.58 (<0.001)
6. Full attention	132	0 (0)	8 (0–10)	0.53 (<0.001)
7. Talking about feelings re: possibility you might get sicker	128	65 (51)	0 (0–10)	0.28 (0.001)
8. Talking about details re: possibility you might get sicker	131	54 (41)	4 (0–10)	0.33 (<0.001)
9. Talking to you about how long you might have to live	132	104 (79)	0 (0–10)	0.10 (0.263)
10. Talking to you about what dying might be like	132	118 (89)	0 (0–10)	0.21 (0.014)
11. Involve you in decisions if get too sick to speak for yourself	129	22 (17)	7 (0–10)	0.38 (<0.001)
12. Asking about things in life that are important to you	131	97 (74)	0 (0–10)	0.43 (<0.001)
13. Asking about your spiritual or religious beliefs	131	114 (87)	0 (0–10)	0.18 (0.041)
Global rating item (GRI)	132	0 (0)	7 (0–10)	n/a

The Pearson correlation coefficients (*r*) between each of items 1–13 and the GRS are presented in Table 3, showing that item 3 (‘answering all your questions about illness and treatment’) had the highest *r* (0.59), followed by item 5 (‘caring about you as a person’), which had a *r* value of 0.58. The *r* values for all of items 1–6 reached statistical significance (*P* < 0.001). It should be noted that within the general communication subscale (items 1–6), there was only a small absolute difference (0.07) between the items with the strongest and weakest correlation coefficients. None of these *r* values were found to be statistically significantly different from one another (*P* = 0.45–0.99). In the EOL‐specific communication subscale, the correlation coefficients are smaller; however, the high proportion of ‘didn't do’ responses associated with these items limits interpretation.

### Qualitative findings

We have previously described the qualitative findings in more depth^11^ and provide only a brief summary here. A total of 16 participants were interviewed, 11 of whom were female (69%) and with a mean age of 78.4 years (SD 11.4). Most participants (*n* = 11; 69%) had a non‐cancer diagnosis. Despite the use of a maximum variation sampling strategy, only Caucasian participants consented to the qualitative interviews. Analysis of the interview transcripts led to the identification of two major themes. The first major theme, ‘*knowing me*’, relates to the influence of life history and social relationships on shaping personal values and healthcare preferences. This theme is further broken down into the subthemes ‘*acknowledging family roles*’ and ‘*respecting one's background*’. The second major theme, ‘*conditional candour*’, describes participants' expressed preferences for receiving frank information from a physician, but with some important qualifications that are elaborated in the subthemes ‘*assessing readiness’, ‘being invited to the conversation*’ and ‘*appropriate delivery of information’*. In Table 4, we provide illustrative quotes for each of these themes and subthemes. Although our focus was on participant perspectives, it is notable that the qualitative data suggest that physicians' level of engagement in EOL communication was at least sometimes well aligned with patients' degree of readiness. In one illustrative case, a female participant described a recent interaction with two physicians while she was in the hospital:

**Table 4:** Qualitative themes: illustrative quotes

*Major theme: ‘knowing me’*
Well it's because they know you, they know what you're like and, you know, it's just like the doctor that saw [my daughter] learning to drive. I mean you could see the fun he got out of thinking “I brought her into the world and now look, she's driving a car.”…It's not self‐pride, it's like a family. It's a continuation. [83 y.o. female with non‐cancer diagnosis]
Subtheme: ‘acknowledging family roles’
And he [doctor] went through it [treatment decision]…and so I said “I'd like to discuss it with my son.” So he made arrangements for that afternoon to be here when my son was in. [82 y.o. female with non‐cancer diagnosis]
Subtheme: ‘respecting one's background’
Well there's sort of a bond or connection between you. If you know someone fairly well it's easier to do things with them, work out plans. But if you're more like a stranger, um they really don't know what you might like or what's best for you and you don't really understand them. [74 y.o. female with cancer diagnosis]
*Major theme: ‘conditional candour’*
Well I think sure, there are times when…people feel that they want the peace of mind knowing what's going to happen to them… [80 y.o. male with non‐cancer diagnosis]
Subtheme: ‘assessing readiness’
I guess [doctor] has to feel around for how much information do I want and then go about, uh, then she has to determine how it's to be presented to me. So, you know, very difficult questions to resolve. [68 y.o. male with cancer diagnosis]
Subtheme: ‘being invited to the conversation’
Just lay it out there and say there's some stuff that showed up on your test or whatever that I'd like to discuss with you if you want to discuss it. And if you're serious about not knowing then we don't have to discuss it. [58 y.o. female with cancer diagnosis]
Subtheme: ‘appropriate delivery of information’
A female came through the door and kind of woke me up, yelled, “I need an answer yes or no [about resuscitation status].” And I said “I'm sorry. I'm just not in a position right now to make a decision.” And she said, “Well I have to know yes or no.” [82 y.o. female with non‐cancer diagnosis]

Interviewer So you mentioned that the doctors were sympathetic. What in particular did the doctors do that you found helpful?

Participant They weren't here for very long but it was just their general attitude that it was something that, you know, needed to be discussed but they didn't want to push it. And it was up to me, you know. They weren't sort of pushing it either way and giving me time to think about it.

(84 y.o. female with non‐cancer diagnosis)

On the other hand, some of the data suggest that physicians neglected such conversations despite patient interest:

Patient No, it's never been ‘You're getting older [name], what would you think you would want to do? And if you get sick what would you like us to do?” Nothing like that…that should be a caring doctor.

(72 y.o. female with non‐cancer diagnosis)

### Mixed methods results

Although items 3 and 5 had the strongest correlation coefficients, all of items 1–6 (the items that participants reported as being consistently performed by physicians) were similarly correlated to the GRS. Thus, two dominant themes emerged in the qualitative strand, but none of the QOC items seemed to emerge as more strongly predictive of patient satisfaction than other items. This represents an obvious divergence between the qualitative and quantitative strands that will be further discussed.

The qualitative theme *‘conditional candour’* provides a possible explanation for the large correlation coefficient between item 3 in the QOC (‘answering all your questions about your illness and treatment’) and the GRS. In ‘*conditional candour*’, some of the data suggest that participants prefer their physician to be forthright with EOL‐related topics, and it can be understood that a physician who is willing to answer questions about illness and treatment options, as outlined in item 3, is more likely to be perceived as providing candid information. However, the qualitative data provided a more complex and detailed account of patient preferences for candid conversation. More specifically, the qualitative findings suggest that patients prefer frank communication that is tempered by a physician's ability to assess patient readiness to engage, to titrate information based on the patient's level of readiness and to deliver information in a sensitive manner and in an appropriate context. These nuanced behaviours were not included in the QOC tool and thus not elicited in the quantitative strand. In addition, we could not measure the strength of correlation for some items in the QOC tool that seem somewhat related to the concept of ‘*conditional candour*’, such as those related to prognostication (items 8–10), because of the large proportion of ‘didn't do’ responses.

The second most strongly correlated item on the QOC relates to a physician's ability to convey care for the patient as a person (item 5), and this may converge with the second qualitative theme ‘*knowing me*’. In this qualitative theme, a physician's personal connection with the patient appears to promote a sense of trust that facilitates fruitful EOL conversations (see Table 4, subtheme ‘*respecting one's background’*).

However, despite this seeming convergence between ‘*knowing me’* and item 5 on the QOC, there are subthemes of this qualitative theme that are not well represented on the QOC tool. Specifically, the theme ‘*knowing me’* includes concepts related to knowing one's background, involving family members in EOL conversations with the physician and understanding the importance of family roles. These specific behaviours are not included as items in the QOC.

Another seeming divergence between the qualitative and quantitative strands relates to the finding that the qualitative data analysis could not provide a substantive explanation for the relatively strong correlation coefficients for items 1, 2, 4 or 6 and the GRS in the QOC (see Table 4). Although interview participants consistently spoke of topics related to ‘*conditional candour’* and ‘*knowing me*’, there was little spontaneous reference to the importance of eye contact, listening or showing full attention.

## Discussion

This novel mixed methods study is well positioned to understand, in a holistic manner and from the patient perspective, the physician behaviours that influence quality of communication at the end of life. The mixed methods analysis provides complementarity as the quantitative strand identifies which behaviours were perceived as being important during EOL communication, and the qualitative strand highlights why these behaviours seemed important and in which contexts. In addition, the merging of quantitative and qualitative data resulted in some divergent findings, most likely because of the unique strengths and weaknesses of each method: the quantitative strand involves a validated questionnaire with a larger number of participants, whereas the qualitative strand involves in‐depth interviews to elicit more nuanced aspects of patient preferences.

The mixed methods results suggest some convergence: both the qualitative and quantitative strands reveal that patients desire candid information exchange with their physician. Many other studies provide supportive evidence of patient preferences for candid EOL communication^30^, ^31^, ^32^ including a discrete choice experiment on chronic kidney disease patients which found that most wanted early and detailed provision of prognostic information and discussion of future healthcare wishes.^33^ Both strands also suggest that patients desire personal connection, or a caring attitude (item 5 on QOC) from their physician, and by mixing the strands, we gain further context that this may promote trust, which facilitates EOL conversations and decision making. However, a caring attitude may also be conveyed by a physician's use of eye contact, by carefully listening to the patient and by providing full attention, which corresponds to items 2, 4 and 6, respectively, on the QOC. The interrelatedness of these behaviours might explain why the statistical analysis reveals little difference in correlation coefficients between the items. Trust in a physician might be promoted by these behaviours in addition to the sense of familiarity and willingness to involve family as discussed in the theme ‘*knowing me’*. Previous work conducted by Chochinov, including the development of the ‘patient dignity inventory’, aimed at facilitating increased understanding of the psychosocial stressors faced by patients with life‐limiting illness, is also based on the assertion of the importance of acknowledging the whole person in EOL care.^34^

We found a general paucity of EOL communication between patients and physicians. Of the 611 patients screened for study inclusion, 117 (19%) did not recall any EOL discussion with a physician, including discussions about resuscitation preferences during the current hospitalization. Although we did not document all of the demographic data for people who were screened but excluded, the screening process involved only seriously ill patients with an estimated 50% risk of mortality in the next 6–12 months, according to previously published criteria.^4^, ^5^ It is noteworthy that these seriously ill patients had no recollection of any form of EOL conversations with a physician, as they can be regarded as a high priority population. Furthermore, there were a large number of ‘didn't do’ responses in the EOL communication subscale of the QOC tool. Again, this is a remarkable finding considering that all participants were seriously and chronically ill. Moreover, the study population experienced an average of 2.4 hospitalizations over the past year, representing multiple critical incidents that could have created an impetus to discuss EOL issues. Nevertheless, this is not a unique result: a previous study that used the quality of communication tool also noted a large number of ‘didn't do’ responses on the EOL subscale,^23^ and other studies have similarly reported a low incidence of EOL conversations between patients and physicians.^35^, ^36^, ^37^, ^38^ In some cases, it appeared that patients were ready to discuss EOL‐related issues whereas the physician avoided these conversations, possibly because of their own lack of readiness or discomfort. On the other hand, it is possible that at least in some cases, physicians did not engage in many of these EOL‐specific behaviours because they sensed patient reluctance to engage. Indeed, the qualitative findings suggest that some physicians were able to gauge the degree of patient readiness and engage patients only to the degree that made them comfortable. In fact, the qualitative theme *‘conditional candour*’ provides an insight that could not be gained without conducting a mixed methods analysis: a ‘didn't do’ response on the QOC tool may not always be a failure as was implied by the coding strategy, put forth by tool authors, that assigns a zero value to ‘didn't do’ responses. Instead, the absence of a specific activity may be a result of careful assessment of patient lack of readiness – arguably a success in terms of patient‐centred EOL communication. This result has implications for future tool development: questions about patient *readiness* or *preference* in discussing a particular issue may be incorporated in addition to rating physician skill in covering different EOL communication topics. The observed variability in readiness to engage in EOL communication aligns with previous studies that suggested that physicians should learn how to appropriately ‘titrate information’^37^ and that patients often display different levels of readiness to engage in EOL communication and documentation of future healthcare wishes.^39^ On the other hand, the literature provides evidence of several physician barriers to initiating EOL conversations, including difficulty with providing a prognosis, lack of time, lack of skill, lack of training, and concern that such conversations may promote patient anxiety, loss of hope or depression.^40^, ^41^, ^42^ Thus, the possibility remains that certain topics were not addressed even when patients may have been interested and ready to engage, as suggested by some of the qualitative data in this study. Future studies could focus on how strongly physicians' EOL communication behaviours align with patient readiness and wishes.

In addition to convergence and complementarity, we also sought to identify divergence between the qualitative and quantitative strands. We identified two dominant themes pertaining to preferred physician behaviours in the qualitative strand and yet none of the QOC items appeared to stand out as behaviours that are much more strongly predictive of patient satisfaction in comparison with other items. This finding, which is a testament to the advantage of the mixed methods design, raises suspicion of a possible ‘halo effect’, in which participants who have a good relationship with their physician might rate all items highly without differentiating between each individual item. The themes *‘knowing me’* and *‘conditional candour’* describe nuanced conceptualizations of patient preferences for EOL conversations with their physician. The richness and complexity of this understanding is difficult to capture on a quantitative tool and may also help to explain some of the divergence between quantitative and qualitative strands.

### Study strengths and limitations

In the Good Reporting of a Mixed Methods Study (GRAMMS) framework, O'Cathain, Murphy and Nicoll suggest criteria by which a mixed methods study can be appraised.^43^ The design, conduct and reporting of this study satisfy the GRAMMS criteria in several ways. Firstly, we clearly stated the rationale for using a mixed methods design: this complex study topic begs for an understanding of not only which behaviours predict patient satisfaction with physician EOL communication, but also why these factors are predictive and in which contexts. Furthermore, we clearly outlined the type of mixed methods design, including the sequence of methods, the equal priority of the qualitative and quantitative strands, the point of interface of the two strands and the way in which data were integrated. We also described and justified the sampling strategies and have highlighted the insights gained by means of mixing methods. In the quantitative strand, we used a validated questionnaire that has been used in previous publications.

One potential limitation of this study is that, because participants endorsed that some specific EOL‐related issues had not been discussed with their physicians, qualitative interview participants were encouraged to share their hypothetical preferences for physician behaviours, and it is not clear how these hypothetical preferences align with what they would actually want. However, this is very similar to the issues encountered in discrete choice experiments, in which participants are asked to make hypothetical trade‐off type healthcare decisions based on their values, and yet discrete choice experiments are considered to be useful for patient‐centred evaluations of health technologies.^37^, ^44^ Another limitation is that only Caucasians agreed to take part in the interviews, despite the attempt to include non‐Caucasians in the qualitative sampling strategy. Similarly, only three participants (2.3%) in the quantitative strand were non‐Caucasians; thus, our findings may not be generalizable to individuals from other cultural groups.

## Conclusion

We sought a more holistic and complementary understanding of patient preferences during EOL conversations by merging results from two strands with different paradigmatic foundations. A patient's sense of familiarity and personal connectedness with a physician, along with a physician's caring attitude and willingness to involve family, may promote satisfaction with EOL communication from the patient perspective. Although candid EOL communication is important, the need to assess patient readiness to engage in conversations, as suggested by the qualitative data, has implications for future quantitative tool development. Specifically, a lack of completion of certain EOL communication‐related tasks does not always signify failure or a missed opportunity but rather a measured approach that takes into consideration patient readiness to engage in the process. Thus, low scores for incomplete tasks are not always appropriate. In addition, the study findings can be used, along with pre‐existing evidence, to aid in the development of EOL communication training curricula for front‐line physicians. The findings may also be used to inform the development of intervention studies aimed at improving EOL communication.

## Contributorship statement

Amane Abdul‐Razzak has been involved in study conception and design, data collection, data analysis and manuscript writing. Diana Sherifali has been involved in study design, data analysis and manuscript writing. John You, Jessica Simon and Kevin Brazil have been involved in data analysis and manuscript writing.

## Data sharing statement

Extra data (including transcribed raw qualitative data and early thematic categorization and coding) is available by emailing Amane Abdul‐Razzak at: amane.abdul-razzak@albertahealthservices.ca.

## Conflicts of interest

There are no potential conflicts of interest, including financial, activities, relationships, affiliations or otherwise, for any of the authors.

## Source of funding

This study was funded by means of a knowledge synthesis grant (grant ID 2013‐RFP2012‐03‐01) through Technology Evaluation in the Elderly, a Government of Canada Network Centre of Excellence Program. This funding agency had no role in design and conduct of the study, data management or analysis, or manuscript preparation, review, approval or decision to submit for publication. Dr. John You is supported by a Research Early Career Award from Hamilton Health Sciences.

## Supporting information


**Data S1.** Quality of Communication (QOC) tool items.
