# Supplementary material for: ‘Talk to me’: a mixed methods study on preferred physician behaviours during end‐of‐life communication from the patient perspective
Source: Health Expect. 2015 Jul 14;19(4):883–96. doi: 10.1111/hex.12384 (PMC5152726; doi:10.1111/hex.12384)
Supplement: Supplementary file 1 — Data S1. Quality of Communication (QOC) tool items. [file HEX-19-883-s001.docx]

Quality of Communication (QOC) tool items^21^

| **General Communication Skills Subscale**  *When talking with Doctor x______ about important issues like becoming very ill, how good is*  *he/she at:*   1. Using words that you can understand. 2. Looking you in the eye. 3. Answering all your questions about your illness and treatment. 4. Listening to what you have to say. 5. Caring about you as a person. 6. Giving you his/her full attention. |
| --- |
| **End-of-Life Specific Communication Skills Subscale**  *When talking with Doctor x______ about important issues like becoming very ill, how good is*  *he/she at:*   1. Talking with you about your feelings concerning the possibility that you might get sicker. 2. Talking to you about the details concerning the possibility that you might get sicker. 3. Talking to you about how long you might have to live. 4. Talking to you about what dying might be like. 5. Involving you in the decisions about the treatments that you want if you get too sick to speak for yourself. 6. Asking about the things in life that are important to you. 7. Asking about your spiritual or religious beliefs. |
| **Global Rating Item**  Overall, how would you rate this doctor’s communication with you about the types of care that you would want if you became sicker or too sick to speak for yourself? |
